# Diagnosis for Latent Tuberculosis Infection: New Alternatives

**DOI:** 10.3389/fimmu.2020.02006

**Published:** 2020-09-10

**Authors:** Claudia Carranza, Sigifredo Pedraza-Sanchez, Eleane de Oyarzabal-Mendez, Martha Torres

**Affiliations:** ^1^Departamento de Microbiología, Instituto Nacional de Enfermedades Respiratorias, Mexico City, Mexico; ^2^Unidad de Bioquímica Instituto Nacional de Ciencias Médicas y Nutrición, Salvador Zubirán, Mexico City, Mexico; ^3^Subdirección de Investigación Biomédica, Instituto Nacional de Enfermedades Respiratorias Ismael Cosio Villegas, Mexico City, Mexico

**Keywords:** latent tuberculosis infection, LTBI, IGRA, TST, LTBI diagnosis

## Abstract

Latent tuberculosis infection (LTBI) is a subclinical mycobacterial infection defined on the basis of cellular immune response to mycobacterial antigens. The tuberculin skin test (TST) and the interferon gamma release assay (IGRA) are currently used to establish the diagnosis of LTB. However, neither TST nor IGRA is useful to discriminate between active and latent tuberculosis. Moreover, these tests cannot be used to predict whether an individual with LTBI will develop active tuberculosis (TB) or whether therapy for LTBI could be effective to decrease the risk of developing active TB. Therefore, in this article, we review current approaches and some efforts to identify an immunological marker that could be useful in distinguishing LTBI from TB and in evaluating the effectiveness of treatment of LTB on the risk of progression to active TB.

## Introduction

Approximately 5–10% of the individuals infected with *Mycobacterium tuberculosis (M. tuberculosis)* develop the disease during the first 2–5 years after infection (1). In the rest of them, the innate immune response will either fully eliminate the infection without leaving a trace of immunological response (resistance to TB infection) (2) or lead to a state of persistent immune response to *M. tuberculosis* antigens without clinical evidence of active disease (2, 3). This last outcome is indeed the basis to consider that one fourth of the world population is infected with *M. tuberculosis* (4). These are the individuals who persist with the so-called latent tuberculosis infection (LTBI) immunoreactivity even if bacterial clearance is achieved, becoming a potential reservoir for active tuberculosis. In this context, there is a clear need of diagnostic assays for LTBI that could be used as well to identify individuals at risk of developing active TB and to monitor response to LTBI treatment.

The identification of individuals in contact with active TB cases during the first 2 years of exposure is important for two reasons: first, because it allows the implementation of public health policies of disease control by identifying individuals with an increased risk of developing the active tuberculosis; in particular, those who acquired the infection recently (4, 5); and second, because it can lead to a better understanding of the immune response during infection, which could be instrumental in the development of better therapeutic and prophylactic interventions.

For a long time, the dominant paradigm for LTBI has been one of a fine balance between host immune response and pathogen metabolism so that progression to active disease occurs whenever this balance is disrupted. However, evidence has been emerging over the past decade that has redefined TB infection in terms of a spectrum of immune responses rather than a static condition (6).

In persons with LTBI, several factors increase the risk of developing active TB. Most of them are related to an impaired immune response, such as concurrent HIV infection, cancer, immunosuppressive therapy or renal transplant, and diabetes. This last condition is particularly important; the incidence of diabetes has been increasing in countries that also have high TB endemicity and because diabetic individuals are approximately three times more likely to develop TB than non-diabetic individuals (7, 8). Some other factors, though, are related to specific components of host response, such as macrophage activation, maintenance of granuloma structure, CD4 T cells, CD8 T cells, interferon-gamma (IFN-γ), and tumor necrosis factor alpha (TNF-α) production, all of them important for the control of the pathogen during LTBI (9). More recently, studies using whole-blood transcriptomic profiling have been performed to identify signatures that could differentiate between LTBI and active tuberculosis and predict different outcomes of treatment (10–12).

In this review, we discuss the strategies aimed at improving the accuracy of diagnosis of LTBI, the possible biomarkers linked to the latency of mycobacterial infection, as well as to the treatment and follow up of patients with a subclinical infection.

## Diagnosis of LTBI

There is no gold standard test for LTBI (3). Indeed, the low tissue bacterial burden associated with LTBI works against any diagnostic strategy focused on the identification of the bacteria or its components. The diagnosis of LTBI is rather indirect and relies on evidence of a cellular immune response to mycobacterial antigens. The most commonly used tests for LTBI diagnosis are the intradermal tuberculin test (TST) and IGRA.

The TST was developed more than a 100 years ago by Robert Koch, also known as “old tuberculin,” or Mantoux test after Charles Mantoux established the diagnosis criteria for reading a TST (13). TST is widely used around the world, in particular in developing countries due to its low cost and straightforward implementation compared to IGRAs. TST has also been used as an epidemiological tool to evaluate the prevalence of LTBI (14). The TST is carried out by injecting intradermal purified protein derivate (PPD) in the forearm of an individual. An induration reaction of 15 mm or larger, read after 48 or 72 h, is considered indicative of past or current mycobacterial infection. The TST requires trained personnel to apply, read, and interpret the test.

TST is based on delayed-type hypersensitivity (DTH) skin reactivation to tuberculin PPD. Tuberculin PPD is a mixture of protein precipitated of mycobacterial culture filtrates, which have undergone modifications (13). There are different manufacturers of PPD referred to as international standard (PPD-SI) and commercial brands under the US FDA standard PPD-S2, such as Aplisol (JHP Pharmaceuticals, Inc, Rochester, MI, USA) or Tubersol (Sanofi Pasteur Limited, Swiftwater, PA, USA). Besides PPD-S2, there are several other formulations such as PPD RT23 produced by Statens Serum Institut, which is the most widely used PPD in the world (13), and a variability of the potency among PPD may affect the TST result.

The question on which components of PPD are mainly responsible for the DTH reaction is an important and unresolved one. Molecular analysis of PPD-S2 has revealed highly conserved chaperone proteins among most of the mycobacterial species such as the 10-kDa chaperonin (GroES, BCG-a heat shock protein), the 60-kDa chaperonin 1 (GroEL), the probable chaperone protein (DnaK, heat shock protein 70), and the heat shock proteins (HspX, alpha-crystallin homolog), which constitute about 60% of the PPD proteomic content (15). Due to the abundance of these proteins, it would be reasonable to expect one or more of them to cause DTH reaction; however, little is known regarding which of them are responsible for it. Moreover, the presence of highly conserved proteins in PPD prevents the TST to distinguish among *M. tuberculosis* infection, Bacille Calmette–Guérin (BCG) vaccination, and exposure to environmental non-TB mycobacteria.

The immune response involved in TST has been the subject of several studies, which had revealed that biological variations among individuals, as those presented below, may explain in part why some persons have strong TST responses, while others present a weak or no response at all.

▪ The CD14 (−159C/T) polymorphism variant (a variant in the CD14 molecule present in monocytes and macrophages), which is associated with a higher probability to be TST negative even if vaccinated with BCG (16).
▪ Th1, Th2, or Th17 immune responses influence TST reactivity: since TST-positive individuals show significantly impaired interleukin (IL)-17 and IL-23 production, lack of Th17 upregulation may be a key feature of TST positivity, while Th2 cytokines could play a marginal role on TST (17). However, IL-17-producing cells are phenotypically and functionally heterogeneous and may play different roles in immune pathology and protection. The role for Th17 during *M. tuberculosis* infection is conflicting and may be protective during acute infection and harmful during chronic infection (18, 19).
▪ A decreased expression of cutaneous lymphocyte antigen (CLA) on skin-resident T cells is associated with later phases of the skin response. A reduced TST reaction in older individuals is not associated to a reduction in the numbers or function of PPD-specific CD4 T cells but to the low expression of CLA, as observed in an *ex vivo* study of the cells associated with the blisters produced by the PPD in the TST (20).
▪ TST anergy may reflect a reduced cell-mediated immune response related to a major codominant gene responsible for TST variability, as suggested by a study of familial segregation on TST reactivity in household contacts of TB index cases in Colombia (21).
▪ A major locus (TST2) on the chromosome region 5p15 controls the intensity of DTH to tuberculin. The absence of TST reactivity has a genetic component and corresponds to the major locus (TST1) on chromosomal region 11p14, which controls TST response and reflect T-cell-independent resistance to *M. tuberculosis*, as per the results of a study in a hyperendemic region in South Africa (22).
▪ Some individuals living in highly endemic areas remain TST negative, suggesting that these individuals are more likely to be naturally resistant to *M. tuberculosis* infection rather than intrinsically deficient in eliciting DTH response (22).

Interestingly, there are other diseases or metabolic states that can also influence the reactivity to TST. Recently, Deniz and colleagues studied 371 patients with chronic kidney disease (which are more susceptible to tuberculosis infection and disease) and found that both high levels of parathormone (PTH) and vitamin D treatment correlate with a negative TST result, indicating that these factors may induce some degree of immunosuppresion (23).

Two interesting reports in children have suggested that helminth infestation may affect the result of immunological tests that evaluate infection with *M. tuberculosis* (24), while the IFN-γ/IL-10 ratio may correlate positively to the TST test, suggesting that the relationship of these two cytokines may be important in TST reactivity (25). This last report also showed that TST is affected by BCG administration but not by exposure to non-tuberculosis mycobacteria (25).

In summary, TST results are affected by a complex array of factors such as age, nutritional and immunological status, the time interval between antigen exposure and the test performance, BCG vaccination, immunosuppression, genetic background, and cross-reactivity with environmental non-tuberculosis mycobacteria and perhaps other pathogens.

The IGRA is a whole blood assay developed in the past decades to detect the IFN-γ produced *in vivo* by sensitized T cells after *in vitro* stimulation with mycobacterial antigens. The mycobacterial antigens used in these assays are the early secretory antigenic target (ESAT-6) and the 10-kDa culture filtrate protein (CFP-10). ESAT-6 and CFP-10 antigens are encoded in the region of differentiation 1 (RD1) present in the *M. tuberculosis* and *Mycobacterium bovis* genome and are absent in the Bacillus Calmette–Guerin vaccine (BCG) and most environmental mycobacteria (26, 27). Therefore, IGRA results are not affected by neither BCG vaccination nor exposure to environmental mycobacteria.

Until 2015, only two types of assays were commercially available: QuantiFERON (QFT) and QuantiFERON TB Gold in tubes (QFT-GIT), which contain long peptides derived from ESAT-6 and CFP-10 (TB7.7, or Rv2654c encoded by RD11 present in QFT-GIT has been removed). QuantiFERON-TB Gold Plus (QFT-Plus), a new generation assay, now includes both long peptides derived from ESAT-6 and CFP-10 (designed to induce a specific CD4 T-cell response) and shorter peptides in an additional tube, to induce IFN-γ production by CD4 and CD8 lymphocytes (28). The inclusion of peptides for stimulation of CD8 T cells has been reported to improve discrimination of LTBI from active TB (29, 30). In general, the QFT-Plus assay demonstrates a stronger association with increased *M. tuberculosis* exposure compared with QFT-GIT in adults with LTBI (28) and, even though both assays correlates well for LTBI diagnosis, the QFT-Plus exhibits a higher sensitivity with similar specificity regardless subject's age (28).

T-SPOT.TB, another commercially available assay, uses the *M. tuberculosis* antigens ESAT-6 and CFP-10. This assay is based on ELISPOT technique, which quantifies the number of IFN-γ-producing T cells (spot-forming cells). It requires expensive reader and software and specialized trained personnel, which restricts its clinical application in developing countries (31). Although The T-SPOT.TB and QuantiFERON assay correlate well, the T-SPOT.TB is less used (32). Considerable discrepancy between TST and T-SPOT-TB test in LTBI individuals has been reported (33).

In addition, different studies have reported a heritability of IFN-γ response to mycobacterial antigens including ESAT-6 and that the percentage of heritability was different in the population assessed, but the higher heritability was described in South African subjects using sibling pairs, and the estimated IFN-γ response heritability was 58% for ESAT-6 (34, 35).

Similar to what has been described for TST, the performance of the IGRA tests can be affected by several factors, mainly related to an impaired immune response and to technical issues. For example, the addition of IL-7 increases test positivity (36). The clinical accuracy of IGRAs seems to be negatively affected in patients with immune-mediated inflammatory diseases (IMIDs) (37) such as Crohn's disease, where the function of immune cells is suppressed (38), as well as in patients on immunomodulatory drugs such as teriflunomide, which exerts an inhibitory effect on T-cell activation, and outcome in QuantiFERON results changing from positive to negative with marked reduction in IFN-γ (39). In addition, high dose of corticosteroids have been associated a high proportion of indeterminate QTF-GIT results in rheumatoid arthritis patients and inflammatory bowel disease. Patients with these conditions, therefore, should be tested with QTF-GIT prior to steroid treatment (40). Interestingly, the IGRA sensitivity is not compromised by diabetes in TB patients; in fact, the sensitivity of QTF was significantly higher in TB patients with diabetes in comparison to those with no diabetes (41). The technical variations that may affect IGRA results include those related to blood sampling (time, volume), tube shaking, incubation or processing delay (cell viability in blood may be affected), incubation duration, analytical errors, and manufacturing defects (5).

## Comparing TST and IGRA for the Diagnosis of LTBI

Although both the TST and IGRA are used in medical practice for the diagnosis of LTBI, they evaluate different parameters of the immune response that are relevant in immunocompetent individuals.

TST performs an *in vivo* assessment of the delayed-type hypersensitivity in response to PPD of the bacilli, and the readout is the size of the skin induration area after 48–72 h. On the other hand, the IGRA test evaluates the cell-mediated immune response *in vitro*, and the readout is based on the level of IFN-γ produced by circulating effector memory cells (42) and the frequency of effector T cells that produce IFN-γ.

The diversity of antigens used in these tests may account for most of the differences in specificity, but genetic diversity and differences in immune response among individuals also affect the performance and results of both tests. Meta-analyses have confirmed that IGRA is more specific in low-risk, BCG-vaccinated individuals (33, 43) and more sensitive in diagnosing *M. tuberculosis* coinfection in HIV-infected patients (44). Discordant results between TST and IGRA are common in individuals with LTBI, but in the case of IGRA (QuantiFERON-GIT), the accuracy of the test may be improved by a longer incubation period with the stimuli and by including IL-2 level measurements (45).

The diversity of immune response in patients with LTBI may also reflect differences in the participation of specific T-cell subsets. For example, an increased number of CD4CD25 high CD39+ cells (regulatory T cells, defined by these and other markers such as FoxP3) has been observed in individuals TST+ and IGRA+, compared to TST+ and IGRA–, suggesting that higher numbers of these cells allow patients to respond to both tests (46). In addition, the correlation of TST and IGRA varies among individuals from settings of high vs. low incidence, perhaps due to the effects of BCG vaccination, exposure to environmental mycobacteria, or the risk of reinfection (47, 48). This is why TST and IGRA results should be interpreted in the context of prevalence and exposure, as has been highlighted by several authors (49, 50).

Interestingly, fluctuations in IGRA results are observed in individuals with high exposure to *M. tuberculosis* such as healthcare workers, suggesting either a poor reproducibility of the assay or a reinfection causing reversion as a result of the continuous exposure to mycobacterial antigens (51). In addition, false conversions are more commonly seen with IGRAs than with TST in low-risk populations (52).

Generally speaking, both tests exhibit similar limitations. For example, their precision is low in immune-compromised individuals being screened for LTBI. This is a crucial constraint, since these individuals are the ones at higher risk of developing TB. Neither TST nor IGRA, QTF-GIT, or QTF are particularly useful in predicting progression to active tuberculosis. Although the newest QTF-Plus looks promising in discerning between LTBI and active TB and between recent and remote acquired TB infection, it needs further validation in both high- and low-risk populations.

Putting all these data into perspective, it is not difficult to understand why the current TST and IGRA cannot meet the requirements for a test that reliably predicts which individuals are more likely to control the infection and who are more likely to progress to active TB.

One strategy to tackle this problem is to build upon current TST and IGRA to develop better tests. One example of this approach is the C-TB, which is a promising hypersensitivity skin test that uses recombinant ESAT-6 and CFP-10 proteins (53). This test is supposed to combine the low cost of TST and the high specificity of IGRA. Another example is the use of different mycobacterial antigens to improve the IGRA test, such as the Esx-1 substrate protein C (EspC; Rv3615c). This is an ESAT-6-like protein that is as immunodominant as ESAT-6 and CFP-10 in people with TB and LTBI and which identifies *M. tuberculosis-*infected persons who do not react to neither ESAT-6 nor CFP-10 (54).

Building on the existing TST and IGRA tests may not be enough, though, given the diversity of factors that can affect them, including individual genetic background. For this reason, alternative strategies have been explored.

## Latency Antigens as Potential for Differentiating LTBI From Active TB

Since LTBI is defined in terms of immunoreactivity to mycobacterial antigens, the selection of the right antigens to evaluate is key in the diagnosis of LTBI and in the development of assays able to discriminate among different states of the infection and the risk of progression to active TB. Numerous studies have focused on the identification of mycobacterial antigens naturally expressed during LTBI.

*In vitro* models of latency combined with genome-wide transcriptome profiling have identified genes that remain upregulated during LTBI and code for proteins known as “latency antigens.”

It should be noted that the term “latency” refers to the state of the, host while the term “dormancy” refers to the state of the bacteria during the latency state. Dormancy is a reversible metabolic shutdown, a state of low bacterial metabolism that is associated to a transition from replicating to non-replicating bacilli, in which cells are able to survive for a long time without replication displaying immune-evading strategies (55, 56). Oxygen deprivation and levels of nitric oxide are examples of factors that favor a low metabolic state.

The dormancy survival regulator (DosR) (also called DosR regulon, DevR, Rv3133c) regulates the initial response of *M. tuberculosis* to hypoxia (57). When DosR is phosphorylated by histidine kinases, it results in the induction of about 48 genes (58). Transcriptional analysis under hypoxic conditions has revealed that, while induction of the DosR regulon is transient, over 200 genes known as enduring hypoxic response (EHR) genes remain induced for a long time, showing more stability than the DosR genes and some overlap with those induced in the Wayne model of hypoxia and nutrient deprivation (59). In addition, some studies have demonstrated that DosR regulon-encoded proteins induce a stronger T-cell response in individuals with LTBI compared to patients with active TB, suggesting a potential use for LTBI diagnosis (60, 61). The accumulated (and sometimes conflicting) evidence that links specific latency antigens with cytokine responses include the following observations:

▪ Individuals with remote LTBI show a significantly higher IFN-γ response to Rv2628 (*M. tuberculosis* latency antigen) than individuals with recent infection, which suggests that responses to Rv2628 may be associated with immune-mediated protection against tuberculosis and could be useful to distinguish recent from remote infection (62).
▪ A short stimulation with Rv2031c induces significantly lower IFN-γ, TNF-α, and IL-10 concentrations in active TB patients compared to household contacts and healthy controls (63). Interestingly, some authors have not found differences in IFN-γ response to Rv2031c between TB, LTBI, and healthy controls (60, 62).
▪ A study that evaluated IFN-γ production in response to latency-associated antigens and EHR mycobacterial antigens (CFP10-1, Rv2031, Rv0849, Rv1986, Rv2659c, Rv2693c, and Rv1737) found that Rv1737 (NarK2) was among the DosR regulon-encoded antigens most frequently recognized by individuals with LTBI (64).
▪ There are significant differences in IFN-γ responses for DosR antigens (Rv1735c, Rv2006, Rv2625c, Rv1996, Rv2032, Rv2629, Rv3126c, Rv0081, Rv2631, Rv3130c, Rv2624c, Rv2007c, Rv2028c, and Rv3134) in healthy household contacts compared with patients with active TB, as reported by a study in whole blood that included a wide range of stage-specific antigens to assess IFN-γ response in a long-incubation assay (65).
▪ Antigens Rv1733c, Rv2029c, and Rv2628 have been reported to increase the concentrations of IFN-γ, Granzyme B, IL-17, and sIL-2 during treatment of active TB (66).
▪ The stimulation of peripheral blood mononuclear cells (PBMC) with Rv1737c and Rv2029c seem to increase the IFN-γ or TNF-α-producing CD4 and CD8 T cells in individuals with LTBI compared to active TB (67).
▪ The use of RV2004 induced a strong proinflammatory response (TNF-α, IL-8, IL-1b, and IL-12) in LTBI individuals compared with active TB and healthy controls (68).
▪ Stimulation with Rv2627c, Rv2629, and Rv2630 induced high concentrations of or TNF-α, IL-6, and IL-10 in patients with active TB. The detection of IL-6, IL-17, and IL-8 in supernatants of cultures with Rv0574c, Rv2630, Rv1998, Rv054, and Rv2028c might confirm the roles of these antigens in inflammation and the pathogenesis of TB (69).
▪ Finally, a meta-analysis that screened a total of 1,533 articles and selected 34 for the final analysis found that among a vast number of different mycobacterial antigens, the Rv0081, Rv1733c, Rv1737c, Rv2029c, Rv2031, and Rv2628, all encoded by DosR, were among the most widely studied and have the highest potential for differentiating LTBI from active TB (70).

## Potential Role of Antibodies in LTBI Diagnosis

A widely held paradigm considers the role of the human antibody response against to *M. tuberculosis* in the protection against TB as marginal (71), at least compared with that offered by cell-mediated immunity. This paradigm has been supported by two kind of observations: the presence of high levels of antibodies in the active form of the disease, suggesting that antibodies do not confer protection (72), and the apparently unaffected risk of TB reactivation of patients receiving rituximab, a human/mouse chimeric anti-CD20 antibody that induces a rapid depletion of normal CD20-expressing B cells (73). Although the presence of antibodies in the serum from patients with active TB has led to the development of commercial diagnostic tests, the World Health Organization has not recommended their use as diagnostic tools on the grounds of suboptimal sensitivity and specificity (74).

More recently, however, our understanding of the role of antibodies specific to mycobacterial proteins has started to evolve. Indeed, emerging evidence suggests that, since the metabolism of *M. tuberculosis* changes over the course of the infection, the expression of immunodominant antigens should reflect these changes, leading in turn to differences in the antibody profile between LTBI and active TB that could be exploited for diagnostic purposes (75).

In support of this hypothesis, we can refer to these observations:

▪ Mycobacterial proteins of 36, 25, and 23 kDa, present in membrane vesicles, have been detected only in sera from TB patients but not in healthy controls, while titters of those antibodies are lower in individuals with LTBI (72).
▪ Vaccination with BCG induces immunoglobulin G (IgG) antibodies against Ag85A that are associated with a reduced risk of developing active TB (76).
▪ In another study on BCG vaccination, LAM-specific IgG antibodies have increased significantly after the first primary and booster doses (77).
▪ Neutrophils and monocytes/macrophages present increased internalization and killing of mycobacteria in the presence of specific antibodies (77).
▪ Specific IgG antibody levels against transmembrane protein Rv1733c are significantly higher in LTBI than in TB patients (78). In contrast, levels of antibodies against other specific *M. tuberculosis* proteins are significantly higher in TB patients than in healthy individuals living in the same endemic areas (78).
▪ High levels of antibodies against ESAT-6, P1c1 (membrane-associated phospholipase C1, Rv2351c), HspX, and TB8.4 (Rv1174c) are detected in the sera from TB patients using immuno-PCR based in ELISA assay (79).
▪ Individuals with established LTBI have higher plasma levels of anti-Rv2626c IgG than in recently infected individuals and patients with active TB (80).
▪ Levels of IgM antibodies against *M. tuberculosis* membrane-associated antigens (MtM), IgA antibodies against alpha crystallin (Acr), and a proliferative T-cell response to both antigens can potentially discriminate between LTBI and active TB disease (81).

## Evaluating the Effect of Drug Therapy of LTBI

A successful treatment of LTBI is an important component in the control of TB at a global level (82). Patients should undergo thorough clinical evaluation to rule out active TB before initiating drug therapy for LTBI, since monotherapy with Isoniazid (INH), the most common frontline therapy for LTBI, would be highly inappropriate in the context of active TB. In addition, the individual risk of reactivation must be balanced against potential risks of developing treatment-related adverse events (82). Therefore, patients with recently acquired LTBI should be evaluated for preexisting medical conditions that may increase the risk of such adverse events and should be tested for coinfection with HIV (83).

Although INH significantly reduces the risk of progression to TB, its effectiveness is limited by the need of a prolonged administration (6 and 12 months in immunocompetent and immunosuppressed individuals, respectively) as well as by the associated adverse events (84). In 2018, the World Health Organization issued new guidelines on the treatment of LTBI in children and adults living in countries with high and low incidence rates of TB. The new guidelines include an individualized risk assessment for preventive treatment of high-risk household contacts of patients with multidrug-resistant TB (3).

Although the effectiveness of INH prophylactic treatment for LTBI is weakly established, there is also some evidence of an associated increase in the vulnerability for TB reactivation and reinfection, which suggests a therapy-related immune impairment (85). In this regard, a study performed in a murine model of mycobacterial infection showed that, although treatment with INH decreases the number of bacteria, INH-treated mice present a more profound suppression of antigen-specific proliferative response compared to untreated mice, even when INH reduces the number of bacteria (85). Based on the above, it is clear that individuals at risk of TB should undergo a clinical evaluation before starting LTBI treatment and that the elimination of TB requires developing new, safer drugs for the treatment of LTBI that can be administered for shorter periods, as well as adequate biomarkers to assess the efficacy of LTBI treatment.

Assessing the effectiveness of LTBI therapy is not straightforward, since infected individuals have no symptoms and mycobacteria cannot be isolated. This has led to the search of immunological markers for this purpose (86). However, one important challenge intrinsic to LTBI is the great diversity of associated physiological and clinical conditions. While some individuals can harbor lifelong chronic non-progressive infection, others present some degree of transient immunodeficiency (e.g., related to other conditions such as HIV, diabetes, or immunosuppressive therapy) that allows LTBI to progress, without an effective treatment and monitoring, to an active form of the disease. Such diversity in the immune status may correlate with a diversity of granulomas containing dormant mycobacteria (6) and could be responsible, in part, for the differences observed in the response to drug therapy.

Since the diagnosis of LTBI is established on the basis of the immune response of individuals to mycobacterial antigens, the immunological tests used for the diagnosis have also been adopted to assess response to drug treatment. This assessment includes measuring IFN-γ in response to mycobacterial antigens, since IFN-γ is considered to be one of the prominent surrogate markers of protective immunity against *M. tuberculosis* (9, 87). So far, few studies have evaluated the effect of INH therapy on the immune response of people with LTBI, and these studies have yielded conflicting results (88–90). Such conflicting data could be associated to variables like the prevalence of TB, antibiotics used, treatment adherence, type of assay (QuantiFERON varieties vs. TB-SPOT), incubation periods (short vs. long), and antigen types (proteins vs. peptides).

In addition, not all studies agree on the target antigen. While studies using ELISPOT have focused on the response to ESAT-6, others have concentrated on the response to CFP-10 (91–93).

All in all, there is still no convincing evidence of the reliability of neither the IFN-γ levels nor the numbers of IFN-γ-producing cells in assessing the response to INH treatment, a view that is consistent with other authors (90, 94, 95).

The limitations of IGRAs have prompted proposals to measure several biomarkers as a mean to distinguish between patients with active tuberculosis and LTBI. Some of these proposals include the following:

▪ Using chemokine IP10 instead of IFN-γ (96)
▪ Measuring sCD14 or sMD2 levels in plasma, which are higher in active TB than in LTBI (97, 98)
▪ Monitoring a reduction in CXCL10 levels in plasma, which should decrease after 2 weeks of treatment of active TB (99) (perhaps not applicable in LTBI)
▪ Determining overexpression of CCL4 in lung tissue in patients with late-stage TB (100).
▪ Characterization of polyfunctional CD4 T cells with a higher proportion of bifunctional T cells producing IFN-γ and TNF-α and effector memory phenotype (EM) in response to CFP-10 and ESAT-6 in active TB and LTBI (101)
▪ Characterization of circulating marginal zone B cells (CD19+IgM+CD23–CD27+) and memory phenotypes to distinguish between active TB and end of treatment (102)
▪ Measuring circulating antibodies secreting cells, memory B cells, and antibodies specific to Cut4 (Rv3452) and CFP21 (Rv1984c) lipolytic enzymes antigens, hypothetically associated with reactivation (103).

On the other hand, studies of whole transcriptomics in patients with LTBI or active TB have revealed groups of genes that are over- or underexpressed in these patients. In the light of these findings, specific gene signatures that correspond to certain stages of the disease have been proposed. In addition, transcriptomic studies allow the study of changes in the expression of transcribed genes throughout the duration of therapy, which can in turn lead to the discovery of biomarkers of diagnostic and prognostic value. Some of these studies, performed in blood cells (10, 11), have already shown that one of the main pathways overexpressed during active tuberculosis is the IFN signaling pathway, which includes both IFN-γ and IFN-αβ induced responses (10, 12).

The next step after a candidate gene has been identified by genomics/transcriptomics is to confirm a differentiated expression by PCR. Following this approach, our group has found that USP18, IFI44L, IFT1, and IL12RA genes are overexpressed in LTBI patients treated with INH, while the expression of CCL4, CXCL11, and IFNA genes is reduced during the INH treatment. Based on this finding, we have proposed these genes as potential biomarkers for the monitoring of response to LTBI treatment (104).

## Detecting Progression to Active TB

Infection by *M. tuberculosis* can no longer be understood in terms of two fixed, mutually exclusive categories (LTBI vs. active TB). Instead, it represents a continuous spectrum of states that differ by the degree of interplay between pathogen replication and host resistance. Signs and symptoms are not always a reliable way to detect the evolution to an active disease, as many times they are subtle, pleomorphic, or easily confused with the clinical presentation of many other conditions. Therefore, the search for diagnostic biomarkers for LTBI and active TB must consider parameters relevant to the whole spectrum of the immune response.

The progression of LTBI to active TB is determined by factors related to the bacteria (e.g., strain virulence, inoculum size, etc.), host (e.g., state of immune response, treatment with steroids, and biologic agents such as antibodies against tumor necrosis factor, solid organ or hematological transplantation, HIV infection, age), and environment (e.g., smoking, occupational exposure in health care workers).

In previous works, IFN-inducible genes in neutrophils had been identified as a specific TB signature; 393 transcripts were identified in whole blood from active TB of intermediate and high burden settings, correlating with radiological extent of disease and reverting to that of healthy controls following treatment (10). TB signature consisting of both IFN-γ and type I IFNαβ signaling genes, contained in the 393 differentially expressed transcripts. Type I IFN-αβ-inducible transcripts has been described as having a dual role: they participate in bacterial control, but they also are associated to an increased susceptibility to TB, as suggested by the direct correlation between its levels in whole blood and the severity of the disease (10). Furthermore, it has been proposed that IFN-α signaling could be valuable to define predictive biomarkers of LTBI progression to active TB (11). The IFN signature comprises a series of genes that are overexpressed in active tuberculosis but revert during the first week of successful therapy, adopting the pattern seen in LTBI (10, 105). In addition, some data suggest that IL-15 and vitamin D levels differentiate active TB from LTBI (106).

Interestingly, in patients with LTBI, but not healthy or BCG-vaccinated individuals, neither patients after TB treatment presented a CD4 cell subset, which is CD27^−^PC-1^+^; these data were interpreted as evidence of *in vivo* induced cell differentiation driven by *M. tuberculosis* antigens, which suggest that these membrane markers could help to differentiate individuals with LTBI from healthy individuals and help to monitor TB drug treatment (107).

Another PPD-specific CD4 T-cell subset secreting TNF-α, but not IFN-γ or IL-2 and with a differentiated effector memory phenotype (CD45RA^−^CCR7^−^CD127^−^), was shown useful to distinguish active TB from LTBI patients (108). In a recent study in which blood cells from patients with active TB or LTBI were stimulated with PPD or ESAT-6/CFP-10, as a result of this stimulation, the CD4^+^CD27^−^CCR4^+^ T-cell subset was induced higher in subjects with active TB compared to those with LTBI. This may indicate that CD27 and CCR4 expression should be investigated as promising immunodiagnostic markers for TB (109).

A recent candidate-gene association study of polymorphisms has focused on ULK1, which encodes a component of an upstream protein complex that transduces signals to central autophagy effectors. ULK1 has been associated with susceptibility to LTBI in Asian individuals; rs12297124 minor allele, a non-coding ULK1 single-nucleotide polymorphism, conferred 80% reduction of LTBI. In addition, it was observed that the replication of *M. tuberculosis* is increased in ULK1-deficient monocytes, which in turn is associated with a decreased TNF-α response to stimulation with Toll-like receptor (TLR) ligands and an impaired autophagy (110).

A recent blood transcriptomic study on sorted memory CD4 T cells, aimed at detecting differentiated gene expression signatures between LTBI and uninfected individuals, found a 74-gene signature related to exposure to *M. tuberculosis*. This gene signature reflected the expansion of CD4 T cells subset containing TB-specific peptide reactivity in LTBI (111). Combining transcriptomic data with single-cell protein profiling and *in vitro* stimulation with Ag-specific peptide pools has allowed the identification of a TB-specific CD4 CD62L^−^GPA33^−^ subset of TH1 cells. However, this observation has been limited to the LTBI and healthy population, and therefore, no conclusion can be drawn on whether this profile could be useful in assessing long-term protection against active TB (111).

In addition, a study demonstrated that the frequency of purified protein derivate-specific CD4 T cells secreting TNF-α but not IFN-γ or IL-2 with a differentiated effector memory phenotype (CD45RA^−^CCR7^−^CD127^−^, TNF-α-only T_EFF_) was able to distinguish between active TB from LTBI and correlate with a risk factor for progression (108). Moreover, the proportion of TNF-α-only T_EFF_ with an effector memory phenotype CD45RA^−^CCR7^−^CD127^−^ is significantly higher during recently acquired LTBI in comparison to remotely acquired LTBI, and the phenotype of TNF-α-only T_EFF_ has been associated with a progression risk and active TB in immunocompetent adults (112). During TB disease and recently acquired LTBI, there seems to be an expansion of a heterogeneous population of immature myeloid-derived suppressor cells (MDSCs) at intermediate stages of cell differentiation (113).

Since several studies have identified a biological heterogeneity of the cell immune subsets associated with status of TB infection, immune cell profiling may look promising at evaluating the risk of developing active TB in LTBI individuals.

Some reports indicate that most of the cellular-mediated immune responses are under the control of microRNAs (miRNAs), key regulators that posttranscriptionally repress the expression of target messenger RNAs (mRNAs) (114). miRNAs are 18–25 nucleotide long, non-coding RNAs that inhibit the translation into proteins by a direct interaction with the protein-specific messenger RNA (114).

Modulation of miRNA expression is induced by intracellular bacteria as a mechanism to survive inside host immune cells (115). Differential expression of miRNAs in cells infected with *M. tuberculosis* suggests that the relevance of some miRNAs as biomarkers for LTBI and LTBI reactivation (116) deserves further exploration. This is supported by several observations:

▪ miRNA-31 expression in children with TB is significantly lower compared to healthy children (117).
▪ has-miR-150 is significantly downregulated, while both has-miR-21 and has-miR-29c are significantly upregulated in active TB patients in comparison to those with LTBI and healthy individuals (118).
▪ miR-146a and miR-155 are well-studied in TB infection models. Thus, downregulation of miR-146a expression in alveolar macrophages of patients with pulmonary TB is associated with disease progression, while the lack of miR-155 expression is associated with an increased susceptibility to *M. tuberculosis* infection (119, 120).
▪ miR-223, which regulates CXCL-2, CCL3, and IL-6, has a critical role in the control of TB in myeloid cells (121).
▪ miR-889 and TNF-like weak inducers of apoptosis (TWEAK, target of miR-889) may be considered as potential biomarkers of LTBI reactivation. In this regard, miR-889 expression is significantly higher in patients with rheumatoid arthritis who also have LTBI compared to those not infected or healthy individuals. By the same token, it decreases significantly in those receiving prophylactic LTBI therapy (122).
▪ miR-155 is significantly decreased in the serum of patients with TB in relation to healthy volunteers, suggesting that inhibition of miR-155 may be closely associated with the development of TB (123).
▪ has-miR-197-3p, has-miR-99b-5p, and has-miR-191-5p are expressed higher in different patterns in the neutrophils of healthy controls vs. that in patients with active TB (124).
▪ miR320, miR204, miR-331, miR-147, and miR-210 expressions in B cells are differentially expressed between TB cases and controls, but the biological relevance of these findings is still uncertain (124).

**Figure 1:**
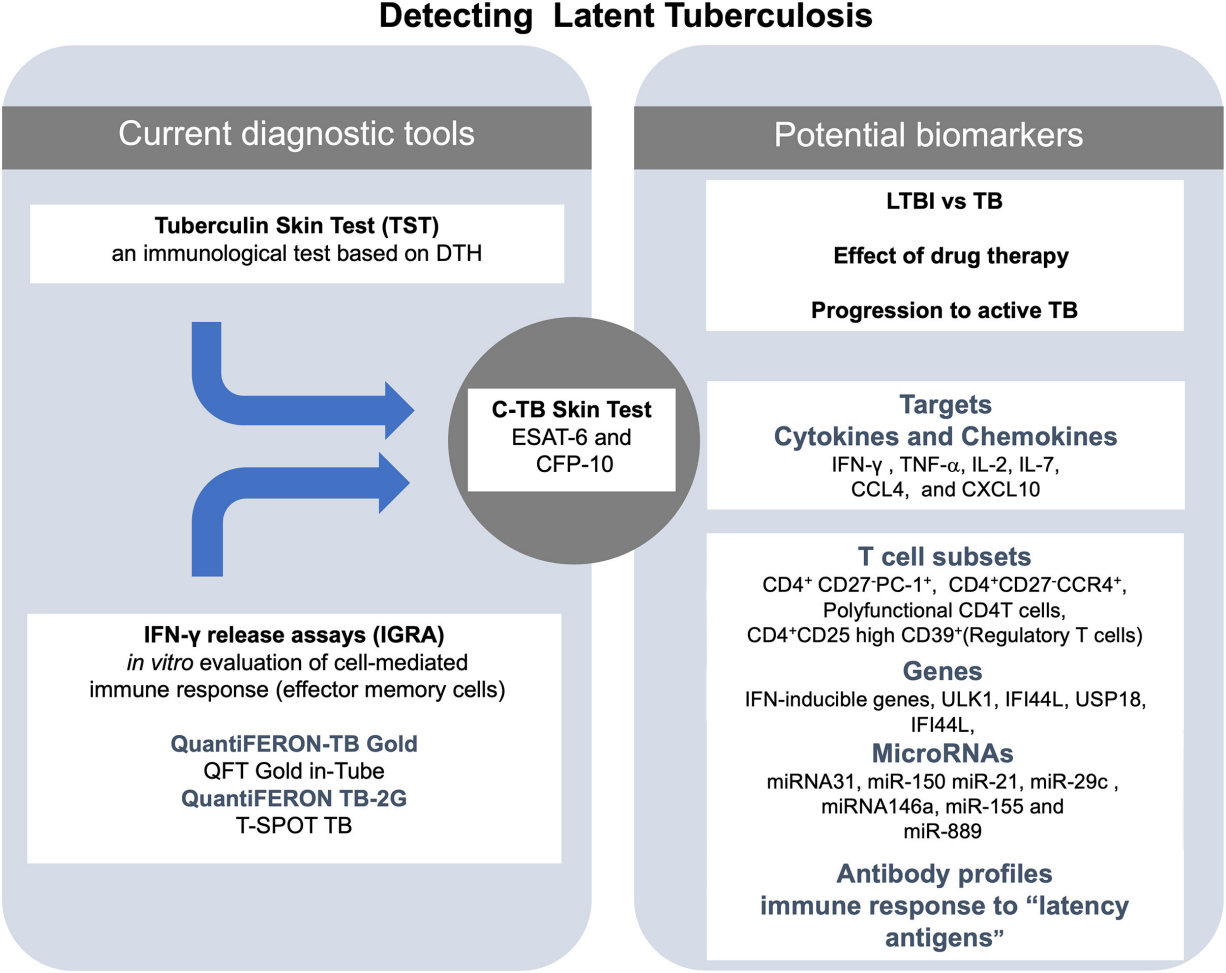
Summary of the actual and potential tools for the diagnosis of latent tuberculosis infection (LTBI). At the center of the figure, there is the C-TB skin test, which combines the sensitivity of the *in vivo* tuberculin skin test (TST) and the specificity of early secretory antigenic target (ESAT-6) and the 10-kDa culture filtrate protein (CFP-10) antigens for *M. tuberculosis* on the left side of the figure are the current diagnostic tools (TST and IGRA). On right side of the figure are listed some of the potential biomarkers to detect LTBI.

In summary, specific miRNAs have shown some power to distinguish between LTBI and active TB. Specifically, evidence rising from multiple independent groups of investigators point out to miR-889, miR223, miR-155, miR-150, miR146a, miR23, and miR-21 as the most promising candidates for this purpose. However, studies with a higher number of individuals to validate these markers are still needed. A general scheme of the laboratory techniques and molecular markers used for diagnosis and treatment of LTBI is shown in Figure 1.

## Concluding Remarks

LTBI represents an occult face of the larger global health problem of TB. A reliable diagnosis and a successful treatment of individuals with LTBI is a paramount issue in the control of TB because they may, eventually, progress to the active form of TB.

The TST has been the most broadly used technique for the diagnosis of LTBI because of its simplicity and the *in vivo* evidence it provides for an antimycobacterial cellular immune response. However, it has the inconvenience of being positive in the BCG-vaccinated individual. The further introduction of IGRAs has added higher specificity, while the new version QTF-Plus looks promising in differentiating between active TB and LTBI. Despite this progress, the search for a reliable biomarker of LTBI and evaluating the efficacy of drug therapy in patients with LTBI remains open.

In this review, we have summarized the main strategies and some targets or immunological markers that have been proposed over the last decade for the differential diagnosis between LTBI and active TB and for evaluating the effectiveness of treatment of LTBI. One of them is the analysis of cellular profile such as the proportion of TNF-α-only T_EFF_ with an effector memory phenotype CD45RA^−^CCR7^−^CD127^−^, which has been associated with a higher risk of progression to active TB in immunocompetent adults. Another is the expansion of a heterogeneous population of immature myeloid-derived suppressor cells (MDSCs), which has been linked to both active TB and recently acquired LTBI. In addition, the cellular response against mycobacterial latency-associated antigens, such as those encoded by the DosR regulon, have been found to be useful in identifying individuals with LTBI or active TB. Other potential candidates include the specific antibody response to distinct *M. tuberculosis* antigens, the identification of specific miRNA, and molecular signatures observed in the analysis of blood transcriptome, such as IFN-γ signaling. The challenges ahead include the validation of these tests in groups of individuals representative of distinct populations and their practicality in low-income countries, where tuberculosis is still a major public health problem. Such challenges, once overcome, may pave the way to a whole new way to deal with the disease.

## Author Contributions

CC, EO-M, and SP-S contributed to the manuscript writing and revision. MT contributed to the manuscript writing and provided the initial idea. All authors contributed to the article and approved the submitted version.

## Conflict of Interest

The authors declare that the research was conducted in the absence of any commercial or financial relationships that could be construed as a potential conflict of interest.
